# Exploring beyond diagnoses in electronic health records to improve discovery: a review of the phenome-wide association study

**DOI:** 10.1093/jamiaopen/ooaf006

**Published:** 2025-02-28

**Authors:** Nicholas C Wan, Monika E Grabowska, Vern Eric Kerchberger, Wei-Qi Wei

**Affiliations:** Department of Biomedical Engineering, Vanderbilt University, Nashville, TN 37240, United States; Department of Biomedical Informatics, Vanderbilt University Medical Center, Nashville, TN 37302, United States; Department of Biomedical Informatics, Vanderbilt University Medical Center, Nashville, TN 37302, United States; Department of Medicine, Vanderbilt University Medical Center, Nashville, TN 37232, United States; Department of Biomedical Informatics, Vanderbilt University Medical Center, Nashville, TN 37302, United States

**Keywords:** electronic health records, phenome-wide association study, phecode, phenotyping

## Abstract

**Objective:**

The phenome-wide association study (PheWAS) systematically examines the phenotypic spectrum extracted from electronic health records (EHRs) to uncover correlations between phenotypes and exposures. This review explores methodologies, highlights challenges, and outlines future directions for EHR-driven PheWAS.

**Materials and Methods:**

We searched the PubMed database for articles spanning from 2010 to 2023, and we collected data regarding exposures, phenotypes, cohorts, terminologies, replication, and ancestry.

**Results:**

Our search yielded 690 articles. Following exclusion criteria, we identified 291 articles published between January 1, 2010, and December 31, 2023. A total number of 162 (55.6%) articles defined phenomes using phecodes, indicating that research is reliant on the organization of billing codes. Moreover, 72.8% of articles utilized exposures consisting of genetic data, and the majority (69.4%) of PheWAS lacked replication analyses.

**Discussion:**

Existing literature underscores the need for deeper phenotyping, variability in PheWAS exposure variables, and absence of replication in PheWAS. Current applications of PheWAS mainly focus on cardiovascular, metabolic, and endocrine phenotypes; thus, applications of PheWAS in uncommon diseases, which may lack structured data, remain largely understudied.

**Conclusions:**

With modern EHRs, future PheWAS should extend beyond diagnosis codes and consider additional data like clinical notes or medications to create comprehensive phenotype profiles that consider severity, temporality, risk, and ancestry. Furthermore, data interoperability initiatives may help mitigate the paucity of PheWAS replication analyses. With the growing availability of data in EHR, PheWAS will remain a powerful tool in precision medicine.

## Introduction

The Phenome-Wide Association Study (PheWAS), akin to an inverted genome-wide association study (GWAS), is a burgeoning biomedical informatics method that can leverage longitudinal electronic health records (EHRs) data to simultaneously investigate numerous phenotypic traits.^1^ PheWAS has the capacity to either replicate known associations or unveil novel links between exposures (eg, genetic variants which are commonly captured through single nucleotide polymorphisms [SNPs] or imputed variants) and diverse diseases, disorders, or other phenotypic traits, such as drugs and their outcomes. At the same time, recent initiatives like the UK Biobank and the NIH All of Us Research Program have provided extensive patient populations with a myriad of phenotypic characteristics, facilitating epidemiological and informatics endeavors.^2^ With these endeavors significantly enhancing the practicality of PheWAS and data extraction, the implementation of PheWAS is becoming increasingly feasible as EHR evolves and data becomes more readily available.

PheWAS has been used across diverse research domains, encompassing gene-disease associations, drug response, gene-environment interactions, and procedure outcomes.^3–5^ Typically, a PheWAS study involves 3 main steps: (1) identification of an exposure of interest, often a genetic variant such as a single-nucleotide polymorphism or a genetic marker previously identified via a GWAS; (2) extraction and transformation of patient data into distinct phenotypes by mapping EHR data to case and control populations; and (3) statistical testing (eg, logistic regression or linear regression) for associations between the exposure variable and clinical phenotypes. Thus, this review investigates the unique types of data leveraged throughout PheWAS analysis. For example, PheWAS often relies on the definition of phenotypes using diagnostic billing or codes such as International Classification of Disease codes (ICD-9 and ICD-10 codes); however, a significant portion of EHR may remain underutilized.^6^ With individual patient profiles containing demographics, medical history, medication information, allergy lists, laboratories and diagnostic test results, immunization history, clinical notes, imaging, vital signs, and billing history, EHRs represent a rich source of data for researchers and PheWAS implementation.^7^^,^^8^

The objective of this review is to summarize previous PheWAS research in the existing literature and to pinpoint potential areas for enhancement by conducting a review of EHR-driven PheWAS. Our study focuses on (1) the types of phenomic data utilized within EHR for PheWAS, (2) the different cohorts or biobanks used for analysis, (3) the size of study cohorts, (4) phenotypes of interest in PheWAS, (5) clinical terminologies utilized, (6) the degree to which studies replicated their findings, and (7) cohort ancestry.

## Methods

### Search criteria

We queried the PubMed database from 2010 (year of the first PheWAS publication) to December 31, 2023. The search terms included keywords relating to PheWAS and informatics approaches found in titles and abstracts (ie, phenome wide association[Title/abstract] OR pheWAS[Title/abstract] OR pheWAS[Title/abstract] OR conceptWAS[Title/abstract] OR medWAS[Title/abstract] OR ddiWAS[Title/abstract] OR proWAS[Title/abstract] OR visitWAS[Title/abstract]). All identified free full-text publications underwent a manual review (performed by N.W.). Our objective was to encompass studies that utilized PheWAS approaches in EHR-based cohorts, exploring topics ranging from genetic-phenotypic associations to drug repurposing and discovery. After our initial search, we filtered data by excluding articles (1) with no full text or abstract availability; (2) PheWAS on cohorts without linkages to EHR^9^^,^^10^; (3) studies focusing on tools and web platforms for PheWAS analysis (these papers were excluded from our analysis but discussed in our results); (4) studies emphasizing GWAS/PheWAS databases rather than PheWAS-related analysis; and (5) meta-analyses, clinical trials, reviews, systematic reviews, randomized controlled trials, editorials, conference abstracts, books, and documents. In instances of decision-making uncertainty, N.W. and W.-Q.W. discussed reaching consensus.

### Data extraction and synthesis

We gathered and organized the following data from each study: exposures (eg, genetic variants, disease traits), phenotypic data (eg, billing codes, clinical text/imaging data, laboratory tests), cohort details, sample size, study objectives (eg, genetic-phenotypic association discovery and drug repurposing), phenotypes of interest categorized by the evaluated physiologic system or characteristic (eg, cardiovascular, dermatologic, and musculoskeletal), associated clinical terminologies (eg, RxNORM, SNOMED, and Phecodes), replication status (as determined through the use of replications cohorts, various ancestral cohorts, or cohorts separated by race), and cohort ancestry. Cohort size was defined as the total number of participants used within the study; in cases where replication cohorts were present, the total cohort size was the sum of the number of participants in discovery and replication cohorts. Cohort size was extracted via manual review and use of Supplementary Information S1. Cohort size was absent in only one of the included articles; this data point was treated as null and reporting bias relating to this missing value was not assessed. Cohort ancestry was categorized using a continental model (eg, groups indigenous to Africa, Asia, Europe, the Americas, etc.). We also mapped ethnicity and race labels to continental ancestry if articles had well-defined distribution data (eg, White British was mapped to European ancestry). The labels “Multi-ancestral” and “Unreported or Ill-defined” were used to imply the presence of multiple ancestries in a dataset and insufficient ancestry reporting, respectively. Data collection was independently performed by N.W., and W.-Q.W. assisted in defining data collection categories. Following collection, we utilized packages in Python for data visualization.

## Results

### Included studies

**Figure 1.**
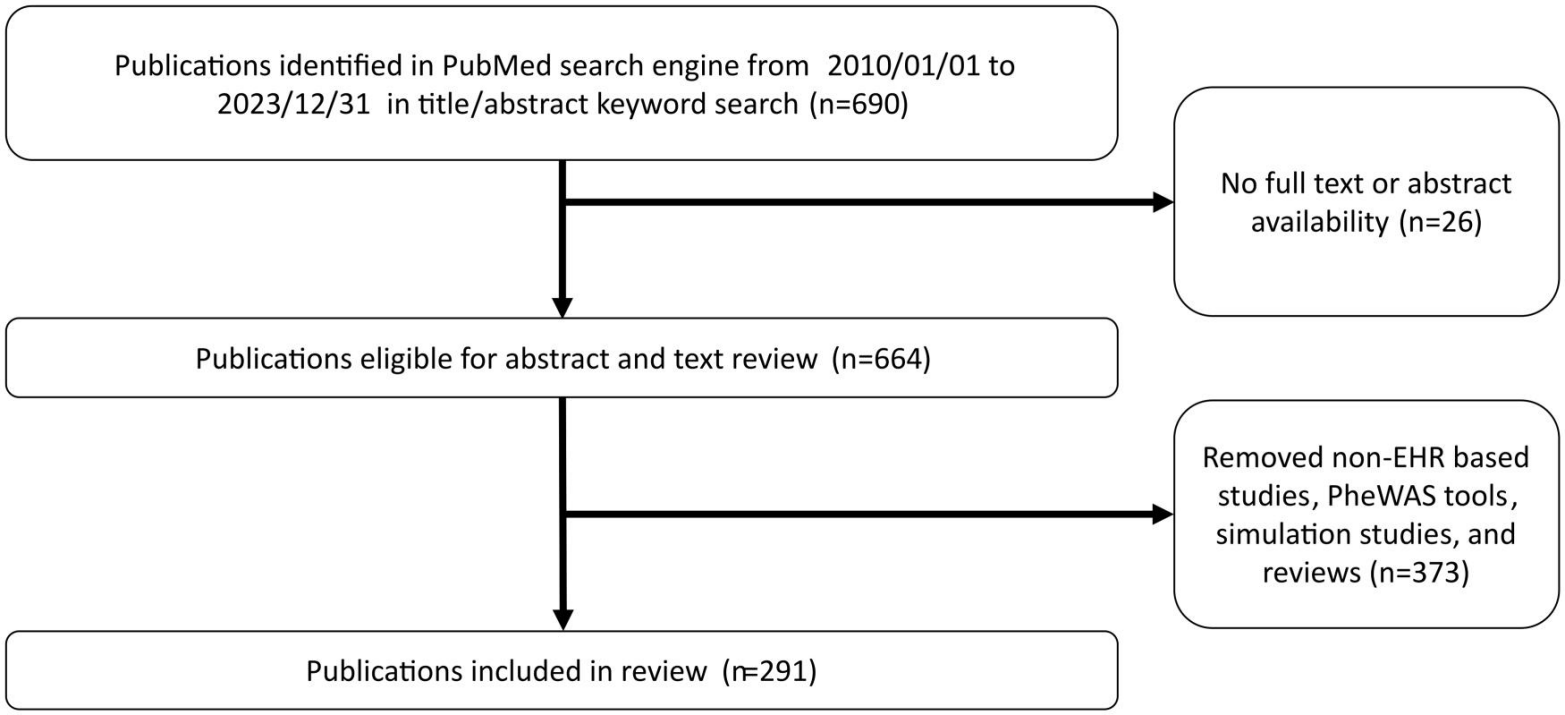
Selection process for relevant PheWAS articles.

We identified 690 articles available in PubMed through our keyword search. After applying our exclusion criteria, 291 papers that involved EHR-based PheWAS and discussed the implications of novel findings and known associations were included in the following analysis (Figure 1). Studies that did not have linkage to EHRs, ie, studies using deeply phenotyped sample cohorts or curated phenotypes from self-report questionnaires, were excluded from the study.^10^^,^^11^ For more information on included studies, see File S1.

### Extracted PheWAS characteristics

Among the 291 evaluated articles, 208 (71.4%) defined their respective phenomes using diagnosis codes, ie, ICD codes or Phecodes. Only 62 (21.3%) articles utilized multiple phenotypic data types. Moreover, 212 (72.8%) studies focused on genetic exposures such as SNPs or polygenic risk scores (PRS) (Table 1).

**Table 1.** Summary of PheWAS article characteristics.

Characteristics		Number of studies	(%)
Phenotyping data	Diagnosis codes	208	(71.5)
	Multiple	62	(21.3)
	Laboratory values	12	(4.1)
	Clinical text	5	(1.7)
	Medications	4	(1.4)
Predictor	Genetic variants/PRS	212	(72.8)
	Disease/disease-related	41	(14.0)
	Biomarkers	15	(5.1)
	Social/environmental risk	11	(3.7)
	Multiple	10	(3.4)
	Medications	2	(0.6)
Population	National biobank	114	(39.1)
	Local biobanks	96	(32.9)
	Multi-cohort	69	(23.7)
	Registry database	12	(4.1)
Size	≥100 000	137	(47.0)
	10 000-100 000	111	(38.1)
	<10 000	42	(14.4)
Terminology	Phecodes	162	(55.6)
	ICD	70	(24.0)
	Multiple	43	(14.7)
	BPA4	4	(1.3)
	CPT	3	(1.0)
	UMLS	2	(0.6)
	Other	5	(1.7)
Replication status	No	202	(69.4)
	Yes	89	(30.5)

Elements of interest were phenotyping data, predictors, cohort, cohort size, and clinical terminologies. Phenotyping data was determined on the basis of data utilized in addition to diagnosis codes as only 5 studies used phenotyping data without diagnosis codes, ie, if a study utilized only diagnosis codes, then it was classified under “diagnosis codes,” and if a study utilized clinical text in addition to diagnosis codes, then it was classified under “clinical text.” If a study utilized more than 2 data types, eg, diagnosis codes, clinical text, and laboratory values, then it was classified under “multiple.” Additionally, “clinical text” encompasses any study using text or image data; though an image is distinct from text, we consider imaging as clinical text as it can be considered vectorized text. Clinical terminology was classified based on terminology utilized for PheWAS implementation. Of note, the “Phecodes” classification, though separate from the ICD category, is based upon ICD terminology mappings to desired Phecodes. Thus, we reported studies as using Phecodes only when article authors stated that they used Phecodes with the PheWAS package or included Phecodes in Supplemental Material S1. Additionally, the “Size” characteristic lacked data from 1 article.

### Trends in published PheWAS

**Figure 2.**
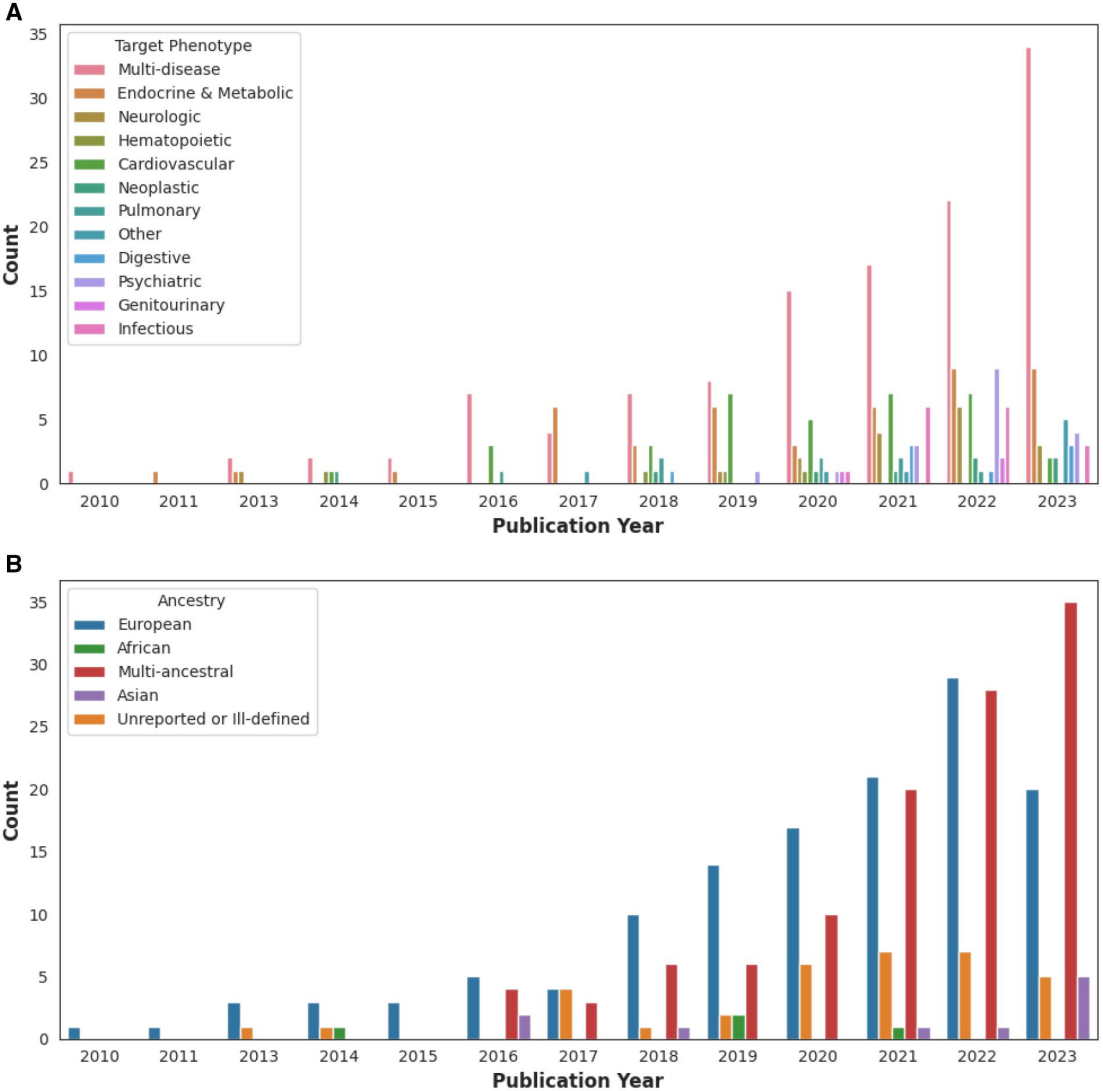
PheWAS phenotypes and ancestry. (A) Phenotypes of interest when performing PheWAS. (B) Cohort ancestry. Of note, the “Multi-ancestral” label was used for UK Biobank PheWAS if ethnicity stratification was mentioned, even if ancestry was not explicitly reported. Additionally, the “Unreported or Ill-defined” label was often used in articles where PheWAS was used as a secondary analysis method and not well described.

Phenotype categories of interest varied across studies, with a plurality (121, 41.6%) focusing on multiple phenotype categories. Additionally, 35 (12.0%) PheWAS studies focus on cardiovascular phenotypes (Figure 2). Common study focuses include coronary artery disease, lipoprotein (a), or PCSK9 inhibitors. More generally, cardiovascular PheWAS tend to focus on comorbidity exploration and genetic marker associations. Similarly, 45 (15.5%) PheWAS studies focused on endocrine and metabolic phenotypes such as nonalcoholic fatty liver disease (NAFLD), systemic lupus erythematosus (SLE), rheumatoid arthritis (RA), and additional autoimmune diseases. At the same time, cohort ancestry was primarily European (45.0%). Apart from a study focused on the Puerto Rican population, multi-ancestral cohorts (38.5%) are also composed of predominantly European or Hispanic subject populations.

**Figure 3.**
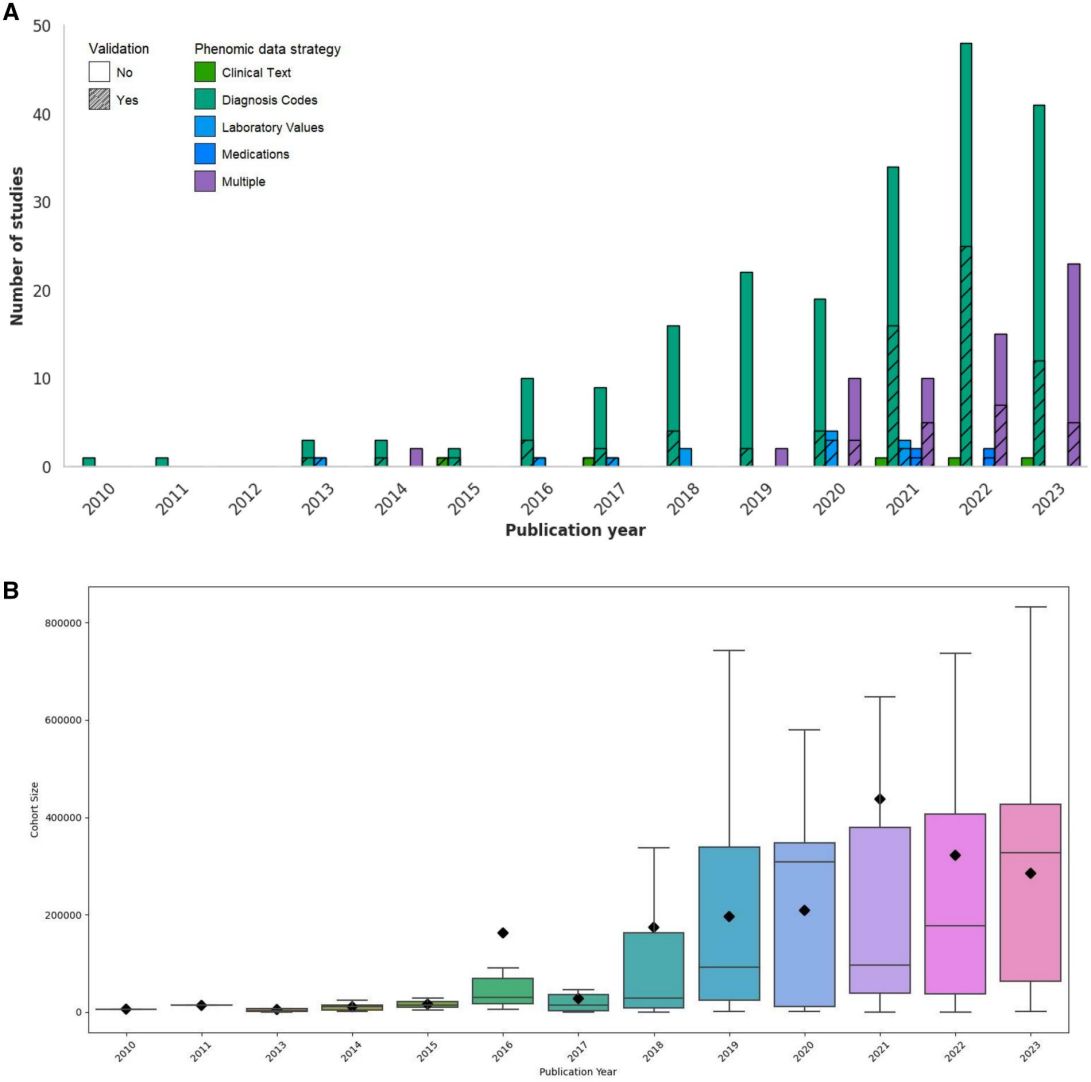
(A) PheWAS publication characteristics over time. Phenotyping data was determined on the basis of data utilized in addition to diagnosis codes, ie, if a study utilized only diagnosis codes, then it was classified under “diagnosis codes,” and if a study utilized clinical text in addition to diagnosis codes, then it was classified under “clinical text.” If a study utilized more than 2 data types, eg, diagnosis codes, clinical text, and laboratory values, then it was classified under “multiple.” Studies were also evaluated on the basis of validation and the frequency of validation was visualized using the diagonally shaded bars. (B) Boxplots corresponding to the cohort size of PheWAS articles in each year. The lower and upper bars represent the first and third quartile values, respectively. Each black diamond represents the mean cohort size from each year. Of note, a PheWAS article from 2023 did not report cohort size so this data point was treated as null for the corresponding boxplot. Additionally, the average cohort size from 2010 to 2013 is 6777.

Since the first PheWAS in 2010, the number of PheWAS studies published annually has grown substantially to over 60 per year in 2023. While diagnosis codes remain the dominant method of defining phenomes for PheWAS, the number of studies leveraging multiple data types, ie, more than 2 of the individual categories, has also increased (Figure 3). The first PheWAS analysis incorporating data extracted from free-text clinical notes was published in 2015, and the first PheWAS using laboratory values was published in 2013.

Population sizes of published PheWAS have also steadily increased, correlating with the increasing availability of EHR data. Studies utilizing between 10 000 and 100 000 participants have been steadily increasing relative to the proportion of studies using less than 10 000 participants. In the same way, the number of studies with over 100 000 participants has also been steadily increasing.

## Discussion

This review highlights the general trends in EHR data usage for PheWAS.

### Are diagnosis codes sufficient for PheWAS? Novel findings and frameworks

Since the advent of PheWAS in 2010, research using PheWAS has yielded many novel findings.^1^ PheWAS has helped in the characterization of pleiotropic effects of genetic variants. For example, a PheWAS study from 2014 in 2 EHR-based cohorts explored pleiotropy in the fat mass and obesity gene (FTO); this study replicated well-described associations between FTO variants and obesity while also identifying novel phenotype associations and demonstrating that pleiotropic effects of the FTO gene may be mediated by obesity.^12^ In addition to strengthening our understanding of pleiotropy, PheWAS has been used with PRS for Alzheimer’s disease to further characterize AD genetic architecture and potential side effects of drugs targeting AD genetic risk factors.^13^ Furthermore, many PheWAS have helped identify or replicate previously discovered genetic variants that contribute to cardiovascular disease, a leading cause of death worldwide.

Though diagnostic coding systems vary across different EHR systems, our findings demonstrate that diagnostic codes remain the dominant tool for PheWAS analysis; for example, less than one third of the reviewed articles did not rely on diagnosis codes or phecodes. However, diagnosis codes, eg, ICD-9 or ICD-10 codes, are primarily used for insurance purposes by medical providers, and therefore, they do not always accurately reflect a true diagnosis.^14^ Moreover, utilizing diagnosis codes for accurate implementation in PheWAS analyses often requires mapping of codes to medical ontologies like phecodes, and ontologies or other phenotyping tools can require further domain expertise as the spectrum of disease varies across different populations.^7^ Use of ontologies optimized for particular biologically relevant patient populations, such as pediatric phecodes (Peds-Phecodes) for analyses of pediatric disease, may yield superior results than using a more general ontology.^15^ As PheWAS analysis aims to address a diverse range of phenotypes across distinct populations and cohorts, the PheWAS design must leverage data beyond diagnosis codes to reflect characteristics, such as severity or temporality, that diagnostic codes cannot capture alone. Furthermore, as many rare phenotypes or population-specific phenotypes may not have diagnosis codes, future PheWAS analysis should consider incorporating deeper phenotyping data. For example, data derived from EHR text and other data structures may better characterize the full spectrum of human diseases by providing more consistent and higher phenotyping performance as compared to diagnosis alone.^6^^,^^16^

With the incorporation of additional data types, researchers have the capability to perform more comprehensive and informative explorations of genotype-phenotype associations. Novel phenome-wide frameworks using clinical laboratory tests (LabWAS) or clinic visit encounters (VisitWAS) can also define robust phenotypes and illuminate phenotypic associations.^17^^,^^18^ Furthermore, frameworks using concepts extracted from free-text clinical notes (ConceptWAS) and semi-structured drug allergy lists (DDIWAS), demonstrate how phenome-wide approaches can characterize emergent diseases and drug-drug interactions, respectively.^19^^,^^20^ In other words, increasingly diverse data types like clinical text have been leveraged in PheWAS with comparable performance to ICD coding^21^ and have been used to address significant challenges like public health and disease progression during the COVID-19 pandemic.^19^ Ultimately, utilizing multiple data types like clinical notes, allergy lists, billing codes, and clinical laboratory results may allow for more comprehensive characterization of the medical phenome and enable discovery of associations that may not be evident through a single data type alone. Additionally, such multi-phenomic data may allow deeper exploration of pleiotropic effects and environmental interactions.

Incorporating multiple data types in PheWAS, however, creates new challenges relating to data integration and interpretation. Leveraging multiple data types increases the number of potential associations that can be examined and creates a higher dimensionality problem, increasing the risk of false-positive associations. Thus, with multiple data types, researchers must optimize multiple hypothesis testing correction methods to maintain statistical power. Within the PheWAS framework, novel statistical tools to optimize multiple-hypothesis testing have been described including Hierarchical Clustering Linear Combination with False discovery rate Control (HCLC-FC), Multivariate Analysis of Variance (MANOVA), the joint model of Multiple Phenotypes (MultiPhen), and Trait-based Association Test that uses Extended Simes procedure (TATES). As emphasized through research on HCLC-FC, statistical methods in PheWAS may aim to: (1) reduce the degrees of freedom of the association tests through clustering, (2) lower implementation cost and runtime, (3) reduce reliance on additional test statistics, and (4) promote generalizability to multiple association frameworks.^22–24^

### PheWAS replication and validation

With the increasing use of PheWAS for exploring the genetic architecture of health and disease, and the potential for dozens or hundreds of significant associations in a single analysis, replication is also essential to provide quantitative evidence that associations are not due to chance or uncontrolled bias in a particular cohort.^25^^,^^26^ Current methods for PheWAS replication typically involve using distinct cohorts from the same biobank or entirely separate biobank or repositories.^27–29^ When replication cohorts are not available, some researchers have opted to perform traditional narrative reviews or pooled analysis to explore the strength of associations.^30–32^ Though not explored in the present study, meta-analysis is an additional tool that can be used to replicate findings and explore consensus among multiple cohorts when characterizing aspects of patient life such as cardiovascular health.^33^ Ultimately, enabling the fast replication of PheWAS may strengthen study power and increase the rate of gene-phenotype discovery. Enabling replication analysis, however, could require increased biobank data collection, improved portability of analytic techniques, more structured and interoperable EHR data, increased sharing of deidentified datasets, or a combination of these efforts.

### Growth of EHR registries and biobanks

While access to patient populations for PheWAS analysis is growing quickly, the size of these patient datasets is also increasing over time (Figure 3). Studies utilizing large-scale registries and biobanks like the Texas Birth Defect Registry, eMERGE, the UK Biobank, or the Million Veteran Program, leverage cohort populations with hundreds of thousands of individuals.^27^^,^^34–36^ Notably, the UK Biobank is the most commonly used data source among the reviewed studies, contributing to over 30% of the studies. With these large-scale analyses, researchers have explored phenotypic trait associations relating to a broad range of factors like social and environmental history to longitudinal comorbidities and biomarkers. More specifically, these large-scale repositories have commonly served as rich sources of socioeconomic data via surveys, laboratory values, diagnostic history, and genetic information.^37–40^ As this trend continues, researchers and physicians will be able to integrate more data via multi-cohort analyses while further refining the PheWAS design.

Despite the growth of biobanks, many PheWAS implementations (45.0%) have restricted their analyses to individuals of European ancestry, and implementations with multi-ancestral cohorts (38.5%) have remained predominantly European or Hispanic, ie, the majority of study participants are of European or Hispanic ancestry in studies containing multiple continental ancestries, with the exception of one study on the Puerto Rican population.^41^ Moreover, several studies (11.7%) have failed to effectively describe cohort ancestry within article text or Supplementary Materials S1, and even fewer PheWAS analyses (4.8%) have solely focused on Asian or African populations. As demonstrated by the extracted ancestries and previous genetic diversity research,^42^ available biobanks and repositories have struggled to capture the full spectrum of ancestries and social groups. With some social groups and ancestries having a historical lack of access to healthcare or distrust with medical systems,^43–45^ the current literature outlines a limitation of the PheWAS design and points to the need for transparency, accessibility, and inclusivity in genetic research.

### Study limitations

Our study has several limitations. Firstly, we focused our literature search on PubMed, which is largely utilized for biomedical literature. Therefore, we may have missed some PheWAS articles discussed in journals from other fields like computer science. In addition, we primarily focused on EHR-driven PheWAS; thus, we did not record characteristics for PheWAS articles that leveraged population/epidemiological data with no linkages to the EHR. For example, studies that utilized phenotypic data from psychiatric interviews or self-reports were not included; importantly, these studies opted to utilize deeply phenotyped cohort data, noting that EHR did not include the full spectrum of desired data.^10^^,^^11^ Finally, we only had a single data extractor which could cause potential biases in data collection, and we did not assess risks of bias within included studies beyond replication status or the use of sensitivity analyses.

### Challenges

In addition to replication, PheWAS researchers also face obstacles with EHR data quality, phenotype coding heterogeneity, and observational confounding. Data quality issues manifest when EHR data is incomplete or inaccurate and can affect the quality of clinical research.^46^ Heterogeneity relates to the diversity of diagnostic coding systems. For example, Brandt et al. identified significant variability in the coding systems used to define phenotype algorithms that can lead to inconsistencies in phenotype definitions.^47^ Sources of observational confounding in PheWAS can include sampling bias in EHR-based datasets or phenotype misclassification within particular data sources.^48^ Additionally, factors such as age of disease onset and disease prevalence in study populations relative to the general population can introduce bias into PheWAS and reduce overall generalizability.^49^ Ultimately, there is a fundamental lack of standardization in defining phenotypes for PheWAS; thus, we advocate for greater transparency in reporting phenotyping algorithms.^50^

High throughput phenotyping tools such as artificial intelligence-based (AI) technologies could also be useful to develop more scalable, reliable, and consistent phenotyping that can be easily transferable to PheWAS application settings. Methodologies in natural language processing (NLP) and generative AI can benefit high-throughput phenotyping by providing efficiently processed, accurate information for PheWAS analysis.^46^^,^^51–53^ As the lack of standardized representation of NLP components in phenotyping algorithms hinders the portability of phenotyping algorithms, it is important to develop interoperable representations that can be used across multiple healthcare systems to support informatics discoveries.^54^ Furthermore, tools such as PLINK, Hail, and PHESANT may be built upon to support multiple medical terminologies as the PheWAS design increases in popularity.^55–58^ These open-source tools and libraries, which provide a range of functionalities from quality control to data manipulation and statistical genetics, are vital for scalable and comprehensive genomic data analysis.

### Future directions

Since the advent of PheWAS, applications of the PheWAS design have been generally hypothesis-free, targeting many phenotypes at once. While only a few physiological systems (eg, cardiovascular, endocrine, and metabolic systems) have been common targets of PheWAS analysis, our study also demonstrates that small proportions of PheWAS analyses have focused on systems such as hematopoietic, genitourinary, musculoskeletal, dermatologic, and injuries. As PheWAS analyses continue to grow more feasible, researchers may aim to address more granular phenotypes of interest that are increasingly prevalent in local populations.

While expanding clinical domain coverage, researchers also have the opportunity to integrate PheWAS analyses with the rapidly emerging standards frameworks for facilitating healthcare and EHR-based data exchange. Current frameworks like the HL7 Fast Healthcare Interoperability Resources (FHIR), the Observational Medical Outcomes Partnership (OMOP) Common Data Model (CDM), the Sentinel CDM, and the PCORnet CDM represent a subset of methods for standardizing vocabularies or terminologies to improve data exchange and analysis.^59–62^

In addition to standardization, researchers must also iterate on the current PheWAS design. For example, the typical PheWAS analysis considers all diagnoses throughout a patient’s longitudinal health record as equivalent in time. Several recent studies have explored how temporal changes in EHR data can be analyzed under the PheWAS framework. A temporal analysis of patients with genetic risk of major depressive disorder (MDD) identifies several diagnoses disproportionately preceded (eg, asthma) or succeeded (eg, chronic pain and substance addiction) a diagnosis of MDD.^63^ Studies from 2 separate EHR cohorts have examined the onset of new medical diagnoses after infection with COVID-19, both of which identified significant increased burdens of new respiratory, circulatory, mental health, neurologic, and pregnancy-related conditions.^64^^,^^65^ Ultimately, continued exploration of temporal changes in longitudinal EHR data using PheWAS may better inform risk prediction and disease pattern recognition, particularly for more temporally variable conditions including infectious diseases, musculoskeletal disorders, or psychiatric disorders.

Ultimately, as the complexity of PheWAS analyses continues to increase, instituting PheWAS reporting standards will be critical. To address issues with replication and generalizability, we suggest adhering to a standardized PheWAS checklist. A checklist may ensure that studies consistently follow best practices and reduce variability. Our suggested checklist includes: (1) study design documentation: clearly define population inclusion and exclusion criteria; (2) study population: specify population size and ancestry along with phenotypic and genetic data sources; (3) phenotype definitions: identify terminologies, ontologies, or algorithms used to define phenotypes; (4) exposure definition: indicate the defined predictor (eg, genetic variants, biomarkers, disease, social risk); (5) statistical analysis: describe statistical methods employed; (6) replication: if possible, outline an approach to replication; (7) data sharing: provide an identifier for the study and ensure that appropriate data and scripts are available for secondary analysis; and (8) ethical standards: confirm that ethical approvals are obtained for all data used. In the end, we also recommend establishing a PheWAS catalog, analogous in scale and utility to the GWAS catalog.^49^^,^^66^ A catalog may serve as a centralized repository, promoting transparency and validation. In the future, researchers may submit study details to a PheWAS catalog, documenting metadata and raw results.

## Conclusion

This review summarizes the general landscape of EHR-driven PheWAS between 2010 and 2023. Recent literature has supported the use of multiple EHR data types to create well-defined phenomes for more robust observational research. Ultimately, further developing PheWAS methodologies that effectively leverage multiple EHR data types while refining methods for extracting phenotypic information from a diverse array of sources will be essential for improving EHR-driven research.

## Supplementary Material

ooaf006_Supplementary_Data

## Data Availability

The collected data and materials used during the current review are all available in this review. Analytic code can also be found in the supplementary materials.
